# The use and misuse of herbarium specimens in evaluating plant extinction risks

**DOI:** 10.1098/rstb.2017.0402

**Published:** 2018-11-19

**Authors:** Eimear Nic Lughadha, Barnaby E. Walker, Cátia Canteiro, Helen Chadburn, Aaron P. Davis, Serene Hargreaves, Eve J. Lucas, André Schuiteman, Emma Williams, Steven P. Bachman, David Baines, Amy Barker, Andrew P. Budden, Julia Carretero, James J. Clarkson, Alexandra Roberts, Malin C. Rivers

**Affiliations:** 1 Royal Botanic Gardens, Kew, Richmond TW9 3AE, UK; 2 College of Life and Environmental Sciences, University of Exeter, Penryn, Cornwall, TR10 9FE; 3 Botanic Gardens Conservation International, Richmond TW9 3BW, UK

**Keywords:** natural history collections, IUCN Red List, conservation assessment, digitization, machine learning, extent of occurrence

## Abstract

Herbarium specimens provide verifiable and citable evidence of the occurrence of particular plants at particular points in space and time, and are vital resources for assessing extinction risk in the tropics, where plant diversity and threats to plants are greatest. We reviewed approaches to assessing extinction risk in response to the Convention on Biological Diversity's Global Strategy for Plant Conservation Target 2: an assessment of the conservation status of all known plant species by 2020. We tested five alternative approaches, using herbarium-derived data for trees, shrubs and herbs in five different plant groups from temperate and tropical regions. All species were previously fully assessed for the IUCN Red List. We found significant variation in the accuracy with which different approaches classified species as threatened or not threatened. Accuracy was highest for the machine learning model (90%) but the least data-intensive approach also performed well (82%). Despite concerns about spatial, temporal and taxonomic biases and uncertainties in herbarium data, when specimens represent the best available evidence for particular species, their use as a basis for extinction risk assessment is appropriate, necessary and urgent. Resourcing herbaria to maintain, increase and disseminate their specimen data is essential to guide and focus conservation action.

This article is part of the theme issue ‘Biological collections for understanding biodiversity in the Anthropocene’.

## 1. Introduction

Global species extinction risk assessments prepared through application of IUCN Red List categories and criteria are increasingly important to monitoring progress against international biodiversity targets [1], informing allocation of conservation resources [2] and guiding business decisions to mitigate biodiversity impacts [3]. Plants, fundamental support systems for life on Earth and the basis for all terrestrial ecosystems, are severely under-represented on the IUCN Red List and increasing their representation is recognized as vital to provide a firm basis for global decision-making, conservation planning and resource allocation [4].

The scale of the challenge is vast. The number of plant species known to science is uncertain, with published estimates varying widely depending on the method of estimation [5], but compared to a recent estimate of *ca* 384 000 vascular plants known to science [6], the 24 057 vascular plant species assessments on the IUCN Red List [7] represent *ca* 6% of known vascular plant species, of which 55% are threatened with extinction.

When assessments other than those on the global IUCN Red List are taken into account, estimates rise to 21–26% of plant species assessed of which 30–44% are assessed as threatened [8,9]. However, assessments of random samples of plants indicate that 21% of species are likely to be threatened [10], suggesting that levels of threat in other compilations are inflated due to threatened species attracting more assessment effort.

Achieving greater and more balanced representation of plants on the IUCN Red List requires assessment of thousands of plant species previously unassessed, especially from the tropics, home to the majority of plant species and subject to greater levels of threat [10]. Despite optimism that the rate of publication of new IUCN Red List assessments for plants was increasing [10], the mean number of plant species added each year remains *ca* 1500, not even sufficient to keep pace with the rate at which plants are described as new to science (more than 2000 per year – average for 2008–2017: 2285 [11]). Furthermore, one in three published plant assessments are outdated, being more than 10 years old.

Calls to increase rates of production of plant extinction risk assessments, whether by IUCN Red List methods or others [12,13], were stimulated by the Global Strategy for Plant Conservation (GSPC), adopted by the sixth Conference of Parties (COP VI) to the Convention on Biological Diversity (CBD) in 2002. GSPC Target 2 called for a preliminary assessment of the conservation status of all known plant species by 2010. However, within a few years there was widespread recognition that Target 2 could not be achieved [14]. Not only was the required working list of all known species still lacking [15,16] but difficulties in assembling data needed for IUCN Red List assessments were daunting [17]. Diverse alternative approaches were proposed and demonstrated, often relying on data from herbarium specimens, then becoming increasingly accessible due to specimen digitization initiatives [18]. In 2010, CBD COP X re-adopted Target 2 along with 14 other targets in more-or-less revised forms for completion by 2020. Scientists continued to propose and refine increasingly sophisticated methods by which the labour-intensive process of assessing plant species extinction risk can be facilitated and expedited, often involving data derived directly or indirectly from herbarium specimens.

Although herbarium specimens have long been recognized as an important source of information for extinction risk assessments [19], many authors have emphasized the problematic aspects of such collections, including biases and errors in taxonomic, temporal and spatial data gleaned from them [20,21]. Despite these limitations, herbarium specimens often represent the best available information for particular species [22]. Their use in assessments of extinction risk is therefore consistent with IUCN Red List Guidelines which expect the assessor to use ‘best available information in combination with inference, suspicion and projection…’ [23].

In this study we review recent use of herbarium specimens in evaluating plant extinction risk and alternative approaches which have been proposed to accelerate this process. We compare performance of a selection of these approaches using five sets of species already assessed by conventional approaches in the past decade. We show that several published approaches can classify species as threatened or not threatened with significant levels of accuracy and that machine learning methods trained on herbarium data rival the best-performing published approaches.

## 2. Use of herbarium specimens in assessments of extinction risk

### (a) Relevance of herbarium specimen information

Limitations of herbarium specimens as data-sources for conservation analyses have been extensively documented in qualitative and quantitative terms over recent years [21,24]. Nonetheless, herbarium specimens have provided the primary or only data source for assessment of extinction risk of thousands of plant species over the same period [25]. In practice, extinction risk assessors do not deny the limitations of specimen data but counter-balance them with the observation that herbarium specimens provide citable and verifiable evidence of the presence of particular species at particular points in space and time. Evidence of this kind is particularly valuable in the context of tropical assessments, for which field observations are often scarce and identifications may be inaccurate due to the exceptional floristic diversity encountered and the dearth of accessible identification aids. Thus, each specimen-based extinction risk assessment represents an evidence-based hypothesis of the current level of extinction risk of a particular plant, to be refined, updated, corrected or refuted as more specimens become available or when the scientific identification of one or more included specimens is corrected or updated in light of new knowledge.

Unlike the taxonomic identification, which may change multiple times over the lifetime of a specimen, other data obtained from herbarium specimens are relatively stable and may be subdivided into (i) ‘metadata’ (objective) recorded at the time of collection; (ii) other narrative (subjective) text added by the collector(s) at the time of collection or label preparation; and (iii) attributes of the actual plant material mounted on the sheet which may be observed and documented. Most specimen databasing projects aim to include metadata, including coarse-level geographical information, collector name, collection number, date, and latitude and longitude coordinates where available [26]. Resources available for data transcription often limit the extent to which narrative information is included in specimen databases, so availability of detailed narrative locality and habitat information is patchy. Finally, information absent from the label but gleaned directly from the specimen is rarely included in major databases [27] and, if captured, may remain inaccessible in the research databases of taxonomists (e.g. morphological details) or ecologists (e.g. phenological observations). All this information is of potential use in extinction risk assessment, either in assigning species to a particular extinction risk or as supporting information for the assessment. However, the degree to which different types of herbarium specimen data are used and cited in assessments varies greatly, reflecting not only their relative utility in the assessment process but also their availability in digital form.

### (b) Use of herbarium data in IUCN Red List assessments

The IUCN Red List categories and criteria were designed to estimate likelihood of extinction risk under prevailing circumstances [28]. The criteria reflect the symptoms of extinction risk such as small, declining or fluctuating populations and small geographical range size [29]. Of the five criteria, geographical range (B) is the most widely applied criterion [30], likely because of availability of suitable data. In 2003, in an early attempt to apply herbarium data to IUCN Red List assessments, a case study based on eight African species reported that herbarium specimen data were most suited to the application of criteria relating to distribution (extent of occurrence, area of occupancy and fragmentation) and population profile/structure (projected continuing decline and number of subpopulations) [19]. Estimates of numbers of locations, numbers of mature individuals, generation length, population size, reduction or fluctuation from herbarium material were considered prone to inconsistencies and subjectivity [19]. To what extent are these conclusions supported by evidence and experience gained over the past 15 years?

#### (i) Spatial data

Spatial data is certainly the herbarium-derived information most widely applied to extinction risk assessments. Most recent specimens (less than 30 years old, since the advent of global positioning systems) include latitude and longitude coordinates, providing a best estimate of the site where the plant was collected, usually within 10s–100s metres depending on equipment used. Older specimens (more than 30 years old) are less likely to include coordinates, but latitude and longitude can usually be deduced from the textual locality information on the specimen, when provided (i.e. through post-collection georeferencing). For very old specimens, where only minimal locality data is reported on the specimen label, the collector's name, number and the collection date can often be combined with itinerary data from published or unpublished journals or travel accounts to infer collection localities. Such labour-intensive approaches for pinpointing older collections may be rewarded by invaluable insights into historic species ranges [31].

Spatial data from multiple herbarium specimens can be collated at species level to provide a distribution map, a required element of supporting information for all IUCN Red List assessments. The distribution data can also be used to calculate two range metrics applicable for the IUCN Red List criteria: (i) extent of occurrence (EOO), usually calculated with a minimum convex polygon around points; and (ii) area of occupancy (AOO), the sum of occupied 2 × 2 km cells. These metrics underpin most plant IUCN Red List assessments (electronic supplementary material, table S1). In addition, distribution maps can be combined with other spatial data layers to inform habitat preference, presence in protected areas, relevant threats, etc.

Inappropriate use of specimen-derived localities to calculate AOO is a common pitfall, which can lead to over-estimation of extinction risk. Sampling densities of herbarium specimens are rarely sufficient to support calculation of AOO using the recommended 4 km^2^ grid cell [23]. A minimum of 500 specimens is necessary to estimate an AOO larger than the AOO threshold for a threatened category (2000 km^2^). As most species are represented in herbaria by only a few specimens [32], their AOO is often underestimated, and could fall within the threshold for a threatened category if AOO is estimated using only herbarium specimens and two of the additional subcriteria are met. A large number of specimens may signify a large number of locations, reducing the impact of AOO underestimation, and potentially pushing a species to a Near Threatened or Least Concern category. However, locations are defined by threatening processes, not always dependent on specimen count or collection locality. Choosing a grid size consistent with sampling density and habitat type of the species is often considered the pragmatic decision for calculating AOO [33], though not strictly consistent with IUCN guidelines.

Both EOO and AOO have also been estimated using other approaches such as species distribution modelling (SDM) [34]. By predicting occurrence based on the environmental attributes of known occurrence, SDMs can ‘fill gaps’ where sampling intensity of herbarium specimens is low. IUCN provides guidance on how SDMs can be applied to estimate EOO and AOO, as the modelled area is often larger than the actual occupied habitat [23]. When using herbarium specimens for SDMs, understanding their limitations becomes particularly important, specifically in relation to numbers of specimens required for modelling, bias in collecting localities and error radii of georeferenced points [23]. All too often, the species excluded from analyses due to sample insufficiency are among the most threatened [35].

Spatial data from herbarium specimens, usually termed ‘locality’ data, is sometimes conflated with the IUCN term ‘locations’, a distinct spatial metric used in the IUCN Red List criteria, which define ‘location’ strictly as ‘a geographically or ecologically distinct area in which a single threatening event can rapidly affect all individuals of the taxon present’ [23]. Location recognition requires identification of plausible threats and analysis of extent of the most serious plausible threat—a step often ignored. Spatial data from herbarium specimens on their own are rarely sufficient to determine threat-defined locations, though notable exceptions include species new to science first collected in areas scheduled for immediate development [36]. However, combining spatial threat datasets with spatial data from herbarium specimens can enable identification of the most plausible threat and resulting threat-defined locations. This step is usually species- or habitat-specific, and can be time-consuming, but for particular plant groups or geographical areas, efficiencies can be achieved when plausible threats are applicable to all species (e.g. wild harvesting of orchids, or harvesting of timber).

Spatial data from herbarium specimens have also been considered for calculation of other spatial metrics such as ‘severe fragmentation’. However, specimen data alone are insufficient to determine severe fragmentation as data on population size, structure and connectivity are required but usually lacking from herbarium specimen labels [23,37].

#### (ii) Temporal data

Temporal data are almost always present on herbarium specimens in the form of collection dates (even if only year, or a range of years for some older specimens) and are usually captured in digitization initiatives. These data are useful for inferring the existence of a particular habitat at a particular point in time. These data can be compared with present day habitat data, such as maps derived from Earth Observations, opening up the possibility of inferring decline of a species' geographical range over time, if present day habitat has been reduced or degraded. By relating the loss of geographical range (EOO, AOO or even quality of habitat) to a decline in the population size of a species, it is possible to apply criterion A if generation length can be estimated [23] or at least to estimate continuing decline as applied in criterion B. Specimen collection dates can also support extinction risk assessments through Population Viability Analysis in which collection dates of the species of interest are interpreted in the context of collection dates of associated species as a proxy for collection effort, facilitating application of criterion E (e.g. [38]). However, no current plant IUCN Red List assessments cite criterion E.

#### (iii) Demographic data

Population size and population decline feature prominently in IUCN Red List categories and criteria. Mathematical models of population dynamics can be incorporated in criterion E to measure the probability of extinction in the wild. However, criterion E and other criteria relying on population data are often not applied because data are not available. Population size is rarely documented on herbarium specimen labels, aside from generalized descriptions e.g. ‘rare’, ‘common’, which are of use as supporting evidence, but not directly applicable to the criteria.

#### (iv) Traits and contextual data

Additional narrative information from specimen labels such as description of the plant (habit), habitat description, uses and threats, although subjective and patchy in availability are of value to the assessor. Phenology information, such as the flowering and fruiting, can also be determined using herbarium specimens. This can be useful in conjunction with size of plants to infer age of mature individuals.

### (c) Errors of omission, inclusion and taxonomic identification

Although increased availability of herbarium specimen data in freely accessible databases has greatly facilitated use in extinction risk assessments it has also given rise to unanticipated issues which merit attention in the hope that they can be avoided in the future.

With rapid growth in the proportion of the world's herbarium specimens represented in databases, such as the Global Biodiversity Information Facility (GBIF), there is a growing tendency to base assessments only on digitally available information (DAI). Analysis has shown that just 10–15 herbarium specimens can be sufficient to produce reliable extinction risk assessments [32] but it should not be forgotten that these analyses were based on well-curated and near-comprehensive collations of extant herbarium material. When relevant material is deposited in herbaria for which digital data are not available, consulting these herbaria to access relevant data remains an important step in ensuring that assessments use the best available evidence. For example, for a range of threatened species in South Africa, provincial herbaria were found to be important sources of data not available elsewhere but drove significant changes to range estimates [39].

Even where all relevant material has been digitized, over-reliance on DAI is a source of error in extinction risk assessments. Relying exclusively on the georeferences in DAI can result in overestimation of extinction risk by omitting specimens which, if georeferenced, would increase estimated range. Without additional georeferencing effort, numbers of species assessed as threatened may be overstated by 50% [17]. In contrast, the opposite may also be true, where extinction risk is underestimated if all specimen records are included uncritically, without checking for and excluding any localities based on cultivated material or from areas no longer likely to support the species (i.e. due to habitat conversion and/or destruction). Exclusive reliance on DAI can also result in propagation of errors due to misinterpretation of label data at the time of transcription. Examination of specimen images, where available, can enable pinpointing and elimination of straightforward transcription errors and georeferencing errors, as well as errors due to misinterpretation of handwritten specimen labels or failure to recognize cultivated specimens.

Another perverse outcome of the rapid growth in herbarium digitization is that many herbarium curators lack resources to maintain digital resources, so that specimen identification records are not updated as they are changed to reflect correction of misidentifications and/or new taxonomic insights. As a result, up-to-date identifications for a taxon of interest often reside not in online databases but on herbarium sheets annotated by specialist monographers as part of their research. While monographic work is often perceived as proceeding at a glacial pace, recent research has shown that cumulated identification changes to specimens over a few years can result in significant changes to extinction risk assessments based on this material [40]. It is therefore vital to ensure that specimen-based assessments include the latest available taxonomic updates.

## 3. Accelerating extinction risk assessments

### (a) Reviewing alternative approaches

Although the IUCN Red List categories and criteria are widely respected, accepted and applied, many scientists report that they are time-consuming to apply and require data that is not readily available [41]. For some, the solution is to develop an independent system tailored to the realities of assessment challenges in their particular context. For example, Mexico's Method for Evaluation of Risk of Extinction for Mexican Wild Species [42] was introduced as a reasonably reliable way to identify species of conservation concern in the face of rapid extinction in a large, extremely diverse country [43]. Similarly, Brazil adopted IUCN categories and criteria, but is developing national microservices-based computational approaches to implement them seamlessly with Brazilian information resources [44]. Here we focus on approaches proposed to facilitate assessment while adhering as closely as possible to IUCN Red List principles, with a view to maximizing consistency of results with those of full IUCN Red List assessments. These ‘IUCN-consistent’ approaches tend to focus primarily on tackling time constraints or limited data availability, though in practice these challenges often co-occur.

#### (i) Time savers

Calculation of EOO and AOO, formerly one of the most time-consuming aspects of IUCN Red List assessment, was transformed by the advent of geographical information systems (GIS). Algorithms first published as the Conservation Assessment Tools (CAT) extension to Arc View 3.3 [45], have been reprogrammed in JavaScript to create GeoCAT, an open source, browser-based tool for EOO and AOO estimation [46] which is exceptionally user-friendly, widely used by botanists and cited over 100 times a year. GeoCAT provides a preliminary estimate of IUCN category based on EOO and/or AOO. This functionality was recently published as an R package rCAT [47]. Another R package, ConR [48], calculates EOO and AOO, and also estimates numbers of locations and subpopulations, creates species maps and offers preliminary estimates of IUCN category based on these results. Tested on herbarium data for African palms previously assessed individually, default settings on ConR correctly classified 71% of species as threatened or not [48].

#### (ii) Less data-intensive approaches

Addressing limitations to availability of data from the world's herbaria, Krupnick *et al.* [49] proposed an algorithm to run on a subset of all herbarium specimens using numbers of verified specimens, the ‘breadth of the localities’ represented and the range of collection dates in a ‘first pass’ to place species in one of three preliminary Red List categories: Potentially Extinct (includes Extinct and Extinct in the Wild); Potentially Threatened (Critically Endangered (CR), Endangered (EN) and Vulnerable (VU)) or Not Threatened (Near Threatened (NT) and Least Concern (LC)). Using Hawaiian plant species well-represented in the Smithsonian Institution's herbarium, their algorithm, later dubbed the ‘US Method’ [17], was calibrated to achieve 95% accuracy in classifying Hawaiian species previously assessed as threatened. The challenge of retrospectively georeferencing large volumes of specimen data prompted the development of the New York (NY) method [17]: EOO is first estimated using existing georeferenced data, then species with EOO estimates exceeding 20 000 km^2^ are tagged as Not at Risk so that retrospective georeferencing efforts are focused on limited numbers of specimens of less common species, for which additional georeferenced data is most likely to influence the extinction risk assessment. When applied to the Puerto Rico flora, the NY and US methods gave results highly congruent with previously completed IUCN assessments, identifying 47 and 42 of the 53 threatened species respectively [17].

#### (iii) Machine learning

Until recently, the lack of high-resolution, high quality occurrence data for most plant species has impeded widespread adoption of machine-learning approaches, despite the success of these powerful, pattern-finding computational techniques in predicting extinction risk for animals [50]. The first report of use of machine learning models to evaluate extinction risk at global scale for a species-rich plant group [51] relied primarily on coarse-scale species distribution data but, for a subset of assessed species for which suitable data were available, models were also tested based on fine-scale range size data, including point data from herbarium specimens. The best such model correctly classified all but one of 81 species (as threatened or not threatened), with range size contributing much more to model performance than when only coarse-scale distribution data were used.

### (b) Comparison of selected approaches

#### (i) Methods

*Approaches selected*. We compared five approaches for predicting threat status of species from herbarium data: (i) Random Forests—using climatic and threat data derived from species ranges; (ii) rCAT—preliminary assessments by the rCAT package [47] based on species' EOO; (iii) ConR—approximate assessments using default settings in the ConR package [48] based on IUCN Red List criteria B; (iv) US Method [49] based on specimen collection data and locality; and (v) Specimen Count—a naive approach based on classifying a species as potentially threatened if the number of specimens is lower than a threshold value (table 1). We defined threat status of each species as either ‘threatened’ or ‘not threatened’ based on its IUCN Red List assessment category; species categorized as CR, EN or VU were ‘threatened’, while species categorised as NT or LC were ‘not threatened’.

**Table 1.** Qualitative comparison of the alternative approaches to identifying threatened species for which quantitative comparisons are a focus of this study.

method/approach	general purpose as stated by authors	more detailed statement of purpose	accuracy (or other metric) reported by authors	data required	skills required	most appropriate metric for evaluating success
ConR [48]	Enable practitioners to conduct preliminary assessments for large numbers of species efficiently	Generate preliminary conservation assessments based on IUCN criterion B that are both reliable and informative	71% of African palm species correctly classified as threatened or not threatened	georeferenced specimen coordinates	basic data handling and knowledge of R	accuracy
Random Forests [50]	Predict the conservation status of Data Deficient species	Determine the true conservation status of DD species without the need for focused field surveys	90% accuracy with 94% sensitivity for a model trained on a global dataset of terrestrial mammals	georeferenced specimen coordinates	geospatial analysis and statistical modelling, knowledge of a programming language	accuracy, sensitivity
rCAT [46]	To calculate EOO and AOO for use in criterion B assessments	Allow practitioners to generate preliminary conservation assessments based on part of IUCN criterion B	unreported	georeferenced specimen coordinates	basic data handling and knowledge of R	accuracy
Specimen Count [52]	Construct a sample of herbarium material as rich as possible in species of conservation concern	Originally proposed as a way of concentrating specimen digitization effort on species most likely to be of conservation concern	82%^a^ sensitivity for a threshold of 10 specimens, tested on endemic legumes of Madagascar	number of herbarium specimens known for a species	basic data handling	accuracy, sensitivity
US Method [49]	Provide a preliminary assessment of the conservation status of plants using data from herbarium specimens	To quickly and accurately identify those species that are *not threatened* i.e. species that are widespread and common and hence can be eliminated from any further analysis	95.1% sensitivity based on plant species of Hawaii, used for calibration; reported numbers correspond to an accuracy between 52.5% and 96.3%	basic specimen information—number of specimens and collection year and/or locality if available	basic data handling and statistical calculations e.g. calculate median	sensitivity

^a^Sensitivity reported is for species of conservation concern, including Near Threatened species as well as Threatened species.

*Validation data preparation*. We tested the different approaches for assessing threat status using species already assessed for the IUCN Red List as a validation set. Data were assembled from five distinct assessment efforts focused on the following plant groups: global *Coffea* (hereafter ‘Coffea’); legumes for the sampled Red List index (‘Legumes’); *Myrcia* sect. *Aulomyrcia* (‘Myrcia’); orchids of New Guinea (‘OrchidsNG’); and Madagascan palms (‘MadPalms’). Species numbers for each group are given in table 2. Information was collated from published global IUCN Red List assessments [7], from completed assessments in the process of submission and publication on the IUCN Red List, and from specimen records underpinning all these assessments. Specimen records had been gathered, as part of the assessment process, from records held at Kew as well as from specialists' databases and aggregators such as GBIF. Duplicate records and those outside a species' native range were removed and coordinates were checked and filled by georeferencing where locality information was available in order to carry out the assessments. After excluding extinct, not evaluated and data deficient species from our dataset, we were left with 1311 species (table 2). All unpublished assessments included in the analysis were reviewed and submitted to the IUCN Red List by October 2018, for processing and publication.

**Table 2.** Groups included in our dataset, with number of species and their distribution.

group	species (specimens) included	total known species	distribution
Coffea	105 (4352)	124	global (confined to Old World)
Legumes	837 (166532)	22347	global
Myrcia (sect. *Aulomyrcia*)	97 (3239)	124	Neotropical
MadPalms	176 (1997)	203	Madagascar
OrchidsNG	96 (1001)	3136	New Guinea endemics
total	1311		

Due to differing requirements, we drew individual sets of predictors (predictor sets) for each approach tested. For Random Forests, we used predictors (features) as described by Bland *et al.* [50] where applicable to plant species and where information was readily available. The US Method required specimen collection year, locality and number of specimens for each species, while both rCAT and ConR required only specimen coordinates, and Specimen Count required only the number of specimens (table 3).

**Table 3.** List of predictors used for each of the compared approaches.

	threat assessment approach						
predictor	rCAT	ConR	US	Specimen Count	Random Forests	short name	type
collection year			✓			—	collection-related
locality	✓	✓	✓			—	collection-related
number of specimens			✓	✓		—	collection-related
genus					✓	genus	taxonomic
family					✓	family	taxonomic
order					✓	order	taxonomic
number of habitats					✓	n_habitats	geographical
biogeographic realm					✓	realm_value	geographical
extent of occurrence (EOO)					✓	range_eoo	geographical
maximum elevation					✓	elevation_max	geographical
minimum elevation					✓	elevation_min	geographical
latitude of range centroid					✓	latitude_centroid	geographical
mean annual temperature					✓	av_temp	climatic
mean temperature seasonality					✓	season_temp	climatic
mean annual precipitation					✓	av_precip	climatic
mean precipitation seasonality					✓	season_precip	climatic
external threat index					✓	eti	threat-related
mean GDP					✓	mean_gdp	threat-related
mean human population density					✓	mean_hpd	threat-related
minimum human population density					✓	min_hpd	threat-related
mean human footprint					✓	mean_hfi	threat-related

Many of the specimens had missing or non-standard locality descriptions. To maximize the number of specimens with locality information for inclusion in the US Method (in which locality is defined at the level of state, province or island ‘depending on regional geography and nationally designated boundaries’), we back-computed the locality for all georeferenced specimens, using the GADM dataset of worldwide administrative areas [53]. Where coordinates were missing, we chose the administrative area that corresponded best to the narrative specimen locality information. The GADM dataset provides a nested hierarchy of administrative units, with the number of levels populated varying between countries. Following testing (electronic supplementary material, table S3), we selected the first level below country (GADM level 1) as representing the best compromise between the stated objectives of the US Method and its published protocol [49].

We removed species from predictor sets for Random Forests and ConR species with ranges spanning the 180th meridian, as these approaches produced infeasible ranges for them. Since each predictor set had differing numbers of species with missing information, different numbers of species remained for each modelling approach (electronic supplementary material, table S4). Although this means that our comparison is not based on exactly the same datasets, this reflects the reality of applying these approaches.

All data collation was undertaken using the Python programming language. For more details of predictors and data-processing see electronic supplementary material.

*Execution of analysis*. We used the entire dataset available to test rCAT, ConR, US Method and Specimen Count. Random Forest classification is a supervised machine learning algorithm, and therefore needs to be trained on a subset of the data. We took 75% of our Random Forest dataset as the training set. We used repeated cross-validation (10-folds, five repeats) on the training set to tune the hyperparameters of the Random Forest classifier, chose the best model based on the area under the receiver operator curve (ROC), and tested the performance of the best model on the remaining 25% of the dataset. We measured predictor importance in the best Random Forest classifier as mean decrease in accuracy by predictor permutation on the out-of-bag samples.

We chose the threshold value for the Specimen Counts approach as the number of specimens that gave the highest accuracy on the whole dataset (electronic supplementary material, figure S1).

*Comparison of threat assessment approaches.* We compared approaches based on their accuracy, sensitivity (correctly predicting threatened species), and specificity (correctly predicting not threatened species). We also compared the accuracy of all approaches to the default accuracy, defined as the accuracy that could be achieved by classifying all species to the most common threat status in the dataset.

We tested differences in value by using Bayesian parameter estimation to model the probability distribution of differences, and we defined a difference as significant when zero was outside of the 95% credible interval of the distribution. We chose a binomial likelihood for differences in accuracy, and a multinomial likelihood for differences in sensitivity and specificity. For ease of computation, we chose flat conjugate priors, and drew 10 000 samples from the modelled posterior distributions. We ran and compared all approaches using R [54].

#### (ii) Results

**Figure 1.**
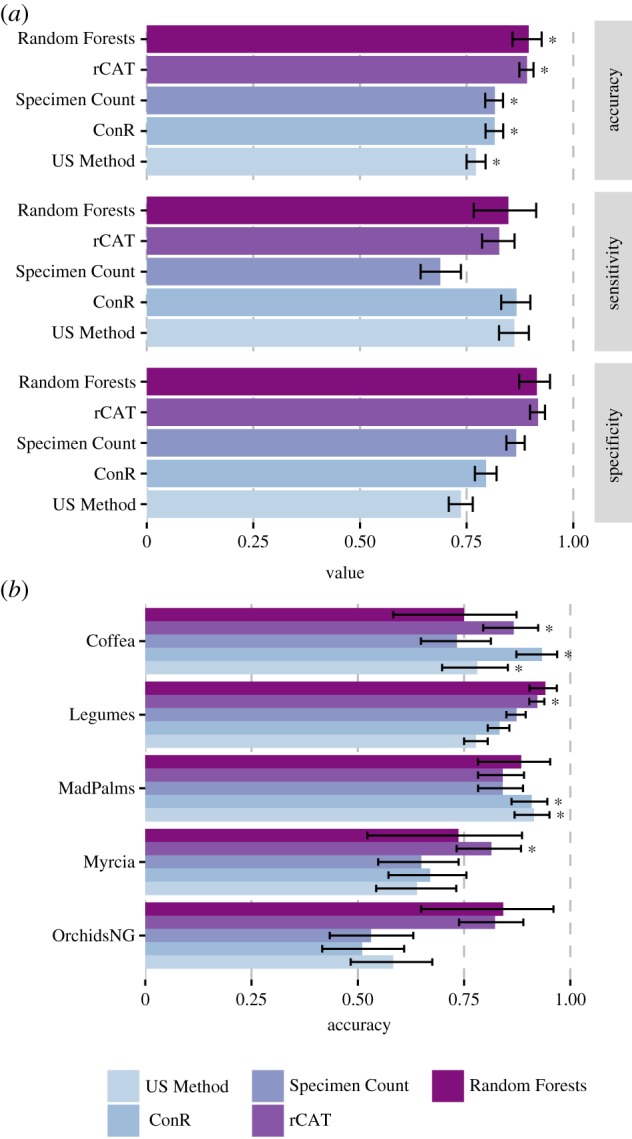
Comparison of each approach (*a*) on the whole dataset by accuracy (correct prediction of threat status), sensitivity (correct prediction of species as threatened), and specificity (correct prediction of species as not threatened) and (*b*) on each group by accuracy. Stars indicate a significant difference from the default accuracy.

*Overview of performance by approach.* Accuracy in correctly classifying as threatened or not threatened species of known extinction risk ranged from 90% to 77% (figure 1*a* and table 4). Across the whole dataset, all approaches achieved accuracies significantly greater than default accuracy but there were significant differences in performance between approaches. Accuracies for Random Forests (90%) and rCAT (89%) were significantly greater than those for Specimen Count, ConR (both 82%) and US Method (77%). Specificity (correctly predicting not threatened species), showed a similar pattern to accuracy, but with ConR and US Method showing significantly lower specificity than the other three methods. ConR achieved the highest sensitivity (correctly predicting threatened species), though not significantly greater than rCAT, Random Forests or US Method.

**Table 4.** Summary of results for each method applied to the test sets overall and to each plant group. Italicized accuracies are significantly better than the default accuracy. Superscript letters indicate significantly better performance than Random Forests (RF), rCAT (RC), ConR (CO), Specimen Count (SC), or the US Method (US).

	group	number of species	accuracy/%	default accuracy/%	sensitivity	specificity
Random Forests	all	326	*90*^CO,SC,US^	72	0.85^SC^	0.91^CO,SC,US^
	Coffea	32	75	62	0.80	0.67
	Legumes	204	94	93	0.64	0.96
	MadPalms	52	88	85	0.98	0.38
	Myrcia	19	74	63	0.67	0.86
	OrchidsNG	19	84	89	1.00	0.82
rCAT	all	1311	*89*^CO,SC,US^	72	0.83^SC^	0.92^CO,SC,US^
	Coffea	105	*87*	68	0.86	0.88
	Legumes	837	*92*	89	0.78	0.94
	MadPalms	176	84	83	0.85	0.8
	Myrcia	97	*81*	62	0.7	0.88
	OrchidsNG	96	82	76	0.96	0.78
ConR	all	1303	*82*^US^	72	0.87^SC^	0.80^US^
	Coffea	105	*93*	68	0.94	0.91
	Legumes	829	83	89	0.73	0.85
	MadPalms	176	*91*	83	0.92	0.83
	Myrcia	97	67	62	0.76	0.62
	OrchidsNG	96	51	76	1.00	0.36
Specimen Count	all	1311	*82*^US^	72	0.69	0.87^CO,US^
	Coffea	105	73	68	0.62	0.97
	Legumes	837	87	89	0.45	0.93
	MadPalms	176	84	83	0.84	0.87
	Myrcia	97	65	62	0.65	0.65
	OrchidsNG	96	53	76	1.00	0.38
US Method	all	1311	*77*	72	0.86^SC^	0.74
	Coffea	105	*78*	68	0.73	0.88
	Legumes	837	78	89	0.75	0.78
	MadPalms	176	*91*	83	0.99	0.53
	Myrcia	97	64	62	0.78	0.55
	OrchidsNG	96	58	76	1.00	0.45

**Figure 2.**
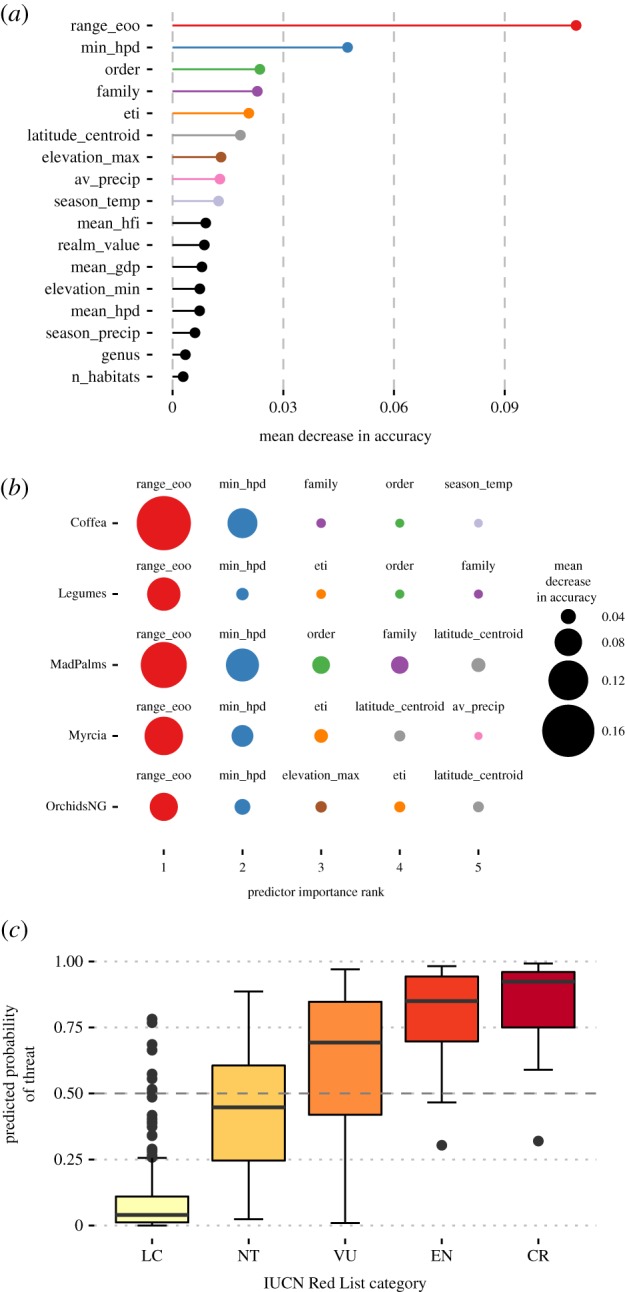
Random Forest classification results: predictor importance measured as mean decrease in accuracy by permutation for (*a*) all predictors and (*b*) the five most important predictors for each group, accompanied by (*c*) the probability of threat by IUCN Red List category predicted by the Random Forest classifier, with the dashed line showing the threshold for classification as threatened. (See table 3 for abbreviations).

Extent of occurrence was the most important predictor in our Random Forest classifier, associated with a mean decrease in accuracy of 11%. Minimum Human Population Density (min_hpd), Order, and Family were next in order of importance, each associated with a 2–5% decrease in accuracy (figure 2*a*).

*Comparison by plant group*. Although all approaches achieved classification accuracies greater than default accuracies for the dataset as a whole, considerable variation was evident between groups (figure 1*b*). At the level of individual groups, rCAT was the most successful approach, achieving accuracies significantly greater than default accuracy in three groups: Coffea, Legumes and Myrcia. ConR and US Method performed best on Coffea and MadPalms. Accuracies greater than default accuracy were not achieved for any group with Specimen Count or Random Forest, nor for any approach applied to OrchidsNG.

Extent of occurrence was the most important predictor for Random Forest classification of each group, associated with mean decrease in accuracy of over 10% for each group, except orchids (8%) (figure 2*b*). Minimum Human Population Density was second in importance in predicting extinction risk in all groups. Environmental predictors were next in importance for OrchidsNG and Myrcia while for other groups Order and Family were more important, as for the whole dataset.

*Comparison by IUCN category and criterion*. The best-performing approaches, Random Forests and rCAT, showed highest accuracy in correctly classifying threat status of LC and CR species (see electronic supplementary material, table S5). Random Forests showed good discrimination for LC and CR species, but had a large overlap in predicted probabilities for VU and NT species (figure 2*c*). ConR matched or exceeded these approaches in accuracy of classifications for CR and EN species but performed less well in classifying species categorized as LC. Accuracies achieved by the US Method were comparable to those of rCAT, Random Forests, and ConR for all threatened categories, but were significantly lower for LC species. All four of these approaches were at their least accurate in classifying threat status of NT species. Naive Specimen Count outperformed other approaches in classifying NT species and, like the best-performing approaches, showed its highest accuracies in classifying threat status of LC and CR species.

IUCN Red List criterion B was the most commonly cited criterion among complete assessments in our dataset and 78% of species assessed as threatened cited criterion B. Criterion D was the next most commonly cited for threatened species at 31%, while criteria A and C were each cited by just 6% of threatened species assessments. Species assessed as threatened citing criterion B had a significantly greater probability of being correctly classified by rCAT and ConR than threatened species not citing criterion B, and species assessed as threatened citing criterion D had a significantly greater probability of being correctly classified by all methods except rCAT than threatened species not citing criterion D. Conversely, species classified as threatened citing criterion A had a significantly lower probability of being correctly classified by any of the five approaches (electronic supplementary material, figure S2).

Further analysis of these performance differences by group revealed that these effects detected in the dataset as a whole were largely driven by differences in performance in Legumes and Myrcia for which all approaches tested except Random Forests had a significantly greater probability of correctly classifying threatened species citing criterion B and a significantly lower probability of correctly classifying threatened species citing criterion A (electronic supplementary material, figure S3).

#### (iii) Overview of comparison results

Both Random Forests and rCAT performed consistently well across our three metrics and did not differ significantly from each other in overall classification accuracy, correct prediction of threatened species (sensitivity) or correct prediction of not threatened species (specificity). Specimen Count and ConR had lower accuracy (82%) and specificity but ConR compared favourably in terms of sensitivity, while Specimen Count had the lowest sensitivity of all methods. The US Method was outperformed by all other approaches in terms of accuracy and specificity but did not differ significantly from Random Forests, rCAT or ConR in terms of sensitivity.

*Unexpected results*. That rCAT, analysing only specimen occurrence data, should match the performance of the much more data-intensive Random Forests approach could be seen as counterintuitive but it may be attributable in part to the fact that most of the completed extinction risk assessments in our dataset were undertaken with the support of GeoCAT, the online tool which uses algorithms almost identical to those of rCAT. Arguably our most surprising result is that the least data-intensive approach, Specimen Count, performed extremely well, matching the classification accuracy of ConR, an approach more comparable to rCAT in terms of data requirements.

*Most important predictors*. Range (EOO) was the most important predictor in our Random Forests model, associated with a mean decrease in accuracy of 11%, similar to results of the only comparable study based on herbarium specimen data [51]. Next in importance in our model were Human Population Density, Order and Family. Population and Family were also recognized as significant correlates of extinction risk in the earlier study [51], in which Order was not considered.

*Best approaches by group*. Drilling down into performance on our different groups revealed significant variation between approaches. The most successful approach at group level was rCAT, with accuracies significantly exceeding default accuracies for Coffea, Legumes and Myrcia, though rCAT accuracy did not differ significantly from Random Forest accuracy for any group. Random Forest results showed all groups had Range (EOO) and Human Population Density as most important predictors.

*Impact of categories and criteria in original assessment*. Discrimination accuracies between threatened and not threatened species were greater for species assessed in categories at the extremes of the scale: CR or LC. Most approaches were least accurate for species assessed as NT, consistent with the fact that this category is the least explicitly based on quantitative parameters [23]. Specimen Count represented a notable exception with 74% accuracy in predicting as threatened species which had been assessed as NT.

Investigating whether performance might vary depending on the criteria on which each original assessment was based, we found that species assessed as threatened had significantly different probabilities of being correctly classified depending on the criteria cited in their assessment. This effect, seen to some extent in all approaches except Random Forests, was particularly evident in Legumes and Myrcia, which may be attributable to the fact that these groups tend to have mean EOO greater than in the other groups (electronic supplementary material, figure S4).

*ConR*. Results for ConR exceeded accuracy levels reported by its creators but were significantly lower than those achieved by Random Forests and rCAT in our study. However, a particular strength of ConR is its high sensitivity, a priority for scientists wishing to minimize the risk that a threatened species is misclassified as not threatened. ConR infers locations from localities and states explicitly an assumption of continuing future decline in habitat quality [55]. Thus, despite statements to the contrary, the resulting preliminary assessments will not be strictly consistent with the theoretical framework provided by IUCN, which requires locations to be defined in relation to the most plausible threat, which will not be habitat decline in all cases. Nonetheless, the breadth of functionality offered by this new R package will likely be of interest to those wishing to extract maximum value from herbarium data for extinction risk assessment.

**Figure 3.**
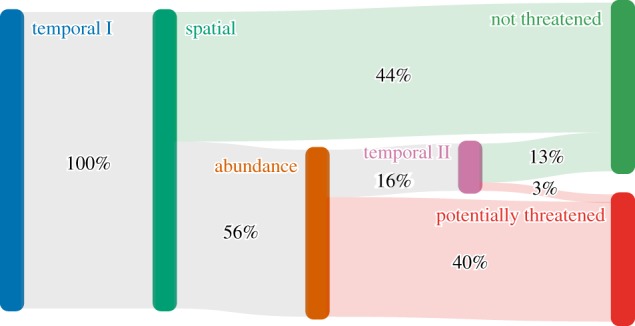
Diagram showing the proportions of species following different pathways towards being classified as potentially threatened or not threatened at each step in the US Method for all plant groups in our study.

*The US Method*. The 77% accuracy achieved by US Method on our dataset is significantly lower than other approaches tested, but it significantly exceeds default accuracy and therefore merits consideration. Sensitivity achieved here with US Method (86%) does not differ significantly from that achieved with other methods, and is markedly better than the 79% reported for its use on the Puerto Rico flora [17]. The US Method is therefore an interesting option for its proposed purpose, as triage for separating species which are clearly not threatened from those meriting more in-depth analysis [49]. A particular strength is its apparent simplicity, likely to appeal to practitioners with limited computing resources or coding skills, who may also appreciate the fact that they can readily visualize the pathway each species follows (figure 3). However, the sensitivity of the US Method is very dependent on the definition of locality used. In our application of the method, following testing, localities were standardized to the first administrative level below country but using finer-grained localities, as also suggested for the US Method, can reduce the sensitivity to 75% (electronic supplementary material, table S3). Given the non-standard nature of specimen locality strings, and the substantial computational effort needed to back-compute and standardize localities as we did, the US Method may prove less simple to apply than it seems. In fact, if sensitivity and simplicity are the key considerations, then the most cost-effective approach to applying the US Method may be to broaden the definition of locality to country (almost always present in herbarium data) at the cost of reducing the specificity (to 55%) and thereby inflating the number of species requiring full assessment.

## 4. Discussion

In our study we have shown that herbarium specimens provide invaluable data for extinction risk assessment of plants; collated and analysed appropriately, they constitute the fundamental resource for increasing the number of plant species that are represented by an evidence-based extinction risk assessment. We also found that several published approaches to accelerating extinction risk assessment of plants offer good discrimination between threatened and not threatened species for a global dataset of tropical and temperate tree, shrub and herb species previously assessed individually following IUCN Red List categories and criteria. Strikingly, the least data intensive approach, simply designating as threatened all species with fewer specimens than a threshold number, showed strong discrimination, offering a useful first step before investing in further collation of data. This is important because the rate at which plants are evaluated for their extinction risk must increase considerably, not only to respond to international targets [1,4,8,14] but also so that plants attract conservation resources proportionate to their importance to life on Earth.

Use of spatial data from herbarium specimens for extinction risk assessment is now widespread and well-established, supported by a range of guidelines, methodological studies and tools which collectively support appropriate use [23,37,46]. Most of the assessments of tropical species added to the IUCN Red List in recent years are based directly or indirectly on herbarium data. Increased DAI from herbarium specimens has been important in extending use of herbarium data for extinction risk assessment, though confining analyses to DAI can markedly reduce assessment accuracy. The increasing disconnect between herbarium specimens and their digital surrogates must be remedied by allocation of sufficient resources for herbaria to update DAI in line with changing specimen identifications. Without such action, ongoing extinction risk assessments based on specimen data will be compromised.

International policies and initiatives to increase the proportion of plant species represented by extinction risk assessments have varied in effectiveness. The CBD's GSPC Target 2 highlighted the low representation of plants, engaged scientists based in herbaria and botanic gardens to a greater extent than ever before, lent a sense of urgency to assessing plant extinction risk using the IUCN categories and criteria and prompted development of technical approaches and tools to accelerate these processes. Despite the failure to meet Target 2 by 2010, lasting positive outcomes include significant engagement in extinction risk assessment by plant taxonomists, many of whom now regularly use extinction risk assessment tools. For example, 84% of recent new species from Brazil were published with extinction risk assessments and 25% of these cite GeoCAT (Canteiro 2018, unpublished data). The Barometer of Life initiative is likely to meet its target for numbers of plants added to the IUCN Red List but has focused on new assessments to the detriment of reassessments which are vital for evaluation of trends and inclusion in international biodiversity indicators [1,4,10].

Our comparison of a selection of more-or-less quantitative approaches to classifying plant species as threatened or not threatened using herbarium data showed that the most data-intensive approaches are not always the most accurate and that effective discrimination may be achieved with surprisingly small quantities of herbarium data. Although more work is needed to test the approaches on a wider range of species assessed in different contexts, the results reported will be useful to guide practitioners in selecting approaches most appropriate for their situation and purpose. For example, if the purpose is broad estimates of overall levels of threat in a country, Specimen Count could be a very useful, quick and informative first step. However, if the intention is to proceed to a full extinction risk assessment for most species, then a more data-intensive approach may be appropriate from the outset. And, of course, if the ultimate objective is to inform and influence conservation action, rather than merely contribute to conservation biology literature, then choice of assessment approach must take into account the requirements of funders and the conservation goal.

Despite the innovative approaches to accelerating plant extinction risk assessment discussed here, it is doubtful whether GSPC Target 2 can be met by 2020 [8] but there can be little doubt that without access to and use of herbarium specimen data we might never achieve our goals. Continuing efforts to generate robust, high quality herbarium specimen-based datasets should be a priority, as this will lead to an increase in the number and accuracy of extinction risk assessments for plants. Although herbarium specimen data are likely to remain central to plant extinction risk assessment for the foreseeable future, their value for this purpose and for broader studies of global change will rapidly diminish without support for ongoing collection activity and updating of digital resources on which so much current specimen use depends.

## Supplementary Material

Electronic supplementary material

## Supplementary Material

List of species

## Data accessibility

The code supporting this article is available via GitHub (https://github.com/barnabywalker/threatened_species_classification_comparison). Species included in our dataset are listed in electronic supplementary material.
