# Supplementary material for: The use and misuse of herbarium specimens in evaluating plant extinction risks
Source: Philos Trans R Soc Lond B Biol Sci. 2018 Nov 19;374(1763):20170402. doi: 10.1098/rstb.2017.0402 (PMC6282085; doi:10.1098/rstb.2017.0402)
Supplement: Electronic supplementary material [file rstb20170402supp1.docx]

Electronic supplementary material

|  |  | **Criteria** | | | | | |
| --- | --- | --- | --- | --- | --- | --- | --- |
| **Method** | **Dataset** | **A** | **B** | **C** | **D** | **E** | **Not cited** |
|  |  |  |  |  |  |  |  |
| **IUCN Red List** | - | 0.06 | 0.28 | 0.03 | 0.10 | 0.00 | 0.52 |
|  |  |  |  |  |  |  |  |
| **Random forests** | Coffea | 0.00 | 0.56 | 0.06 | 0.03 | 0.00 | 0.34 |
|  | Legumes | 0.01 | 0.06 | 0.00 | 0.00 | 0.00 | 0.93 |
|  | MadPalms | 0.00 | 0.54 | 0.08 | 0.65 | 0.00 | 0.15 |
|  | Myrcia | 0.32 | 0.32 | 0.00 | 0.00 | 0.00 | 0.37 |
|  | OrchidsNG | 0.00 | 0.32 | 0.00 | 0.00 | 0.00 | 0.68 |
| **rCAT** | Coffea | 0.00 | 0.69 | 0.02 | 0.02 | 0.00 | 0.29 |
|  | Legumes | 0.01 | 0.10 | 0.00 | 0.00 | 0.00 | 0.89 |
|  | MadPalms | 0.00 | 0.50 | 0.10 | 0.61 | 0.00 | 0.17 |
|  | Myrcia | 0.14 | 0.31 | 0.00 | 0.00 | 0.00 | 0.58 |
|  | OrchidsNG | 0.00 | 0.36 | 0.00 | 0.01 | 0.00 | 0.63 |
| **ConR** | Coffea | 0.00 | 0.69 | 0.02 | 0.02 | 0.00 | 0.29 |
|  | Legumes | 0.01 | 0.10 | 0.00 | 0.00 | 0.00 | 0.89 |
|  | MadPalms | 0.00 | 0.50 | 0.10 | 0.61 | 0.00 | 0.17 |
|  | Myrcia | 0.14 | 0.31 | 0.00 | 0.00 | 0.00 | 0.58 |
|  | OrchidsNG | 0.00 | 0.36 | 0.00 | 0.01 | 0.00 | 0.63 |
| **Specimen Count** | Coffea | 0.00 | 0.69 | 0.02 | 0.02 | 0.00 | 0.29 |
|  | Legumes | 0.01 | 0.10 | 0.00 | 0.00 | 0.00 | 0.89 |
|  | MadPalms | 0.00 | 0.50 | 0.10 | 0.61 | 0.00 | 0.17 |
|  | Myrcia | 0.14 | 0.31 | 0.00 | 0.00 | 0.00 | 0.58 |
|  | OrchidsNG | 0.00 | 0.36 | 0.00 | 0.01 | 0.00 | 0.63 |
| **US Method** | Coffea | 0.00 | 0.69 | 0.02 | 0.02 | 0.00 | 0.29 |
|  | Legumes | 0.01 | 0.10 | 0.00 | 0.00 | 0.00 | 0.89 |
|  | MadPalms | 0.00 | 0.50 | 0.10 | 0.61 | 0.00 | 0.17 |
|  | Myrcia | 0.14 | 0.31 | 0.00 | 0.00 | 0.00 | 0.58 |
|  | OrchidsNG | 0.00 | 0.36 | 0.00 | 0.01 | 0.00 | 0.63 |

**Table S1.** The proportion of all plant species listed on the IUCN Red List citing the different IUCN Red List Criteria, followed by a breakdown of species assessments citing each criterion by plant group in the data sets used for each method.

## **Calculation of predictors**

### *Random Forests Predictors*

For random forests, we used the predictors as described by Bland *et al*. [1] where applicable to plant species and where the information was readily available. Information for some of these predictors was sourced directly from the Red List assessments, while other predictors were calculated by extracting values based on the species’ geographic range (Table S2). For the latter, we calculated a species’ geographic range as the minimum convex polygon (MCP) of the mapped specimens associated with its Red List assessment. We then combined the range map and raster layers for the relevant predictor variables and extracted the mean, minimum, and maximum values of the predictor. When extracting these values, we reprojected all ranges and rasters into the equal area Mollweide projection, and buffered ranges to half the resolution of the raster in question to account for cells partially within the species’ range. We calculated the external threat index (ETI) as the mean threat index of every other species occurring in a particular species’ range, weighted by the overlap of their ranges [1, 2]. The threat index was derived by converting IUCN Red List threat categories to a 0 to 5 scale, where 0 = Least Concern, 1 = Near Threatened, 2 = Vulnerable, 3 = Endangered, 4 = Critically Endangered and 5 = Extinct in the Wild/Extinct.

| Predictor | Threat assessment method | | | | | Source | Citation |
| --- | --- | --- | --- | --- | --- | --- | --- |
|  | **rCAT** | **ConR** | **US** | **Specimen Count** | **Random Forests** |  |  |
| Collection year |  |  | ✓ |  |  | Specimen data |  |
| Locality | ✓ | ✓ | ✓ |  |  | Specimen data |  |
| Number of specimens |  |  | ✓ | ✓ |  | Specimen data |  |
| Genus |  |  |  |  | ✓ | Red List |  |
| Family |  |  |  |  | ✓ | Red List |  |
| Order |  |  |  |  | ✓ | Red List |  |
| Number of habitats |  |  |  |  | ✓ | Red List |  |
| Biogeographic realm |  |  |  |  | ✓ | Red List |  |
| Extent of occurrence (EOO) |  |  |  |  | ✓ | Red List |  |
| Maximum elevation |  |  |  |  | ✓ | Red List |  |
| Minimum elevation |  |  |  |  | ✓ | Red List |  |
| Latitude of range centroid |  |  |  |  | ✓ | Calculated from range |  |
| Mean annual temperature |  |  |  |  | ✓ | Extracted from raster | [3] |
| Mean temperature seasonality |  |  |  |  | ✓ | Extracted from raster | [3] |
| Mean annual precipitation |  |  |  |  | ✓ | Extracted from raster | [3] |
| Mean precipitation seasonality |  |  |  |  | ✓ | Extracted from raster | [3] |
| External threat index |  |  |  |  | ✓ | Calculated from range |  |
| Mean GDP |  |  |  |  | ✓ | Extracted from raster | [4] |
| Mean human population density |  |  |  |  | ✓ | Extracted from raster | [5] |
| Minimum human population density |  |  |  |  | ✓ | Extracted from raster | [5] |
| Mean human footprint |  |  |  |  | ✓ | Extracted from raster | [6] |

**Table S2.** Summary of predictors used for each approach, together with how they were derived.

The required predictor information sourced from the Red List assessments was missing for some species and we attempted to fill those gaps using various methods. Where the EOO was missing, we calculated the area of the MCP based on the species occurrences. We filled missing values for biogeographic realm by assigning the nearest realm from a reference map [7] and filled missing elevation information from the BioClim elevation raster [3].

Due to insufficient coverage of some of the raster files, some species did not derive values from these raster calculations, and so were removed these from the dataset (8 species). Some species also had infeasibly large ranges, as they had specimens that occurred across the 180^th^ meridian, and so these were also removed from the dataset (6 species).

### *ConR and rCAT predictors*

Both ConR [8] and rCAT [9] only use the coordinates of species occurrences, so we removed any specimens with missing or obviously wrong coordinates (both longitude and latitude at zero, or values of -9999). Further to this, ConR cannot calculate EOO for species that have occurrences spanning the 180^th^ meridian, and so we removed these from the ConR set of predictors.

### *US Method predictors*

The US Method [10] only uses the number of specimens for a species, and the year and location of collection for these specimens. The initial definition of location used by Krupnick *et al*. was which island of Hawaii the specimen was collected from, but when testing the US Method on species found elsewhere they used the state that the specimen was collected in. Given the different granularity of administrative areas in different countries, this leads to a somewhat subjective definition of area.

To maximise the number of specimens with locality information for inclusion in the US Method (in which locality is defined at the level of state, province or island ‘depending on regional geography and nationally designated boundaries’), we back-computed the locality for all geo-referenced specimens, using the GADM dataset of worldwide administrative areas [11]. Where coordinates were missing, we chose the administrative area that corresponded best to the narrative specimen locality information.

The GADM dataset provides a nested hierarchy of administrative units, with the number of levels populated varying between countries. We chose the administrative level based on the idea that the overall aim of the US Method is to minimise the number of wrongly classified threatened species (maximise the sensitivity) and the aim of the spatial step in particular is to judge if a species is widespread or not. To this end, we tested the US Method at GADM levels 0 to 3. While level 0 gave the highest sensitivity, it corresponds to individual countries, so we chose the level that gave the next best sensitivity (level 1) but also provided some sub-division of localities within countries (Table S3).

| **Administr-ative level** | **Group** | **Number of species** | **Accuracy / %** | **Default accuracy / %** | **Sensitivity** | **Specificity** |
| --- | --- | --- | --- | --- | --- | --- |
| **0** | **All** | **1311** | **65** | **72** | **0.89** | **0.55** |
|  | Coffea | 105 | *82* | 68 | 0.83 | 0.79 |
|  | Legumes | 837 | 62 | 89 | 0.79 | 0.59 |
|  | MadPalms | 176 | *91* | 83 | 0.99 | 0.53 |
|  | Myrcia | 97 | 58 | 62 | 0.81 | 0.43 |
|  | OrchidsNG | 96 | 30 | 76 | 1.00 | 0.08 |
| **1** | **All** | **1311** | ***77*** | **72** | **0.86** | **0.74** |
|  | Coffea | 105 | *78* | 68 | 0.73 | 0.88 |
|  | Legumes | 837 | 78 | 89 | 0.75 | 0.78 |
|  | MadPalms | 176 | *91* | 83 | 0.99 | 0.53 |
|  | Myrcia | 97 | 64 | 62 | 0.78 | 0.55 |
|  | OrchidsNG | 96 | 58 | 76 | 1.00 | 0.45 |
| **2** | **All** | **1311** | ***85*** | **72** | **0.82** | **0.86** |
|  | Coffea | 105 | *79* | 68 | 0.73 | 0.91 |
|  | Legumes | 837 | 88 | 89 | 0.68 | 0.90 |
|  | MadPalms | 176 | *92* | 83 | 0.98 | 0.63 |
|  | Myrcia | 97 | *72* | 62 | 0.62 | 0.78 |
|  | OrchidsNG | 96 | 67 | 76 | 0.96 | 0.58 |
| **3** | **All** | **1311** | ***85*** | **72** | **0.75** | **0.89** |
|  | Coffea | 105 | *78* | 68 | 0.68 | 1.00 |
|  | Legumes | 837 | 89 | 89 | 0.64 | 0.92 |
|  | MadPalms | 176 | 85 | 83 | 0.86 | 0.80 |
|  | Myrcia | 97 | *74* | 62 | 0.62 | 0.82 |
|  | OrchidsNG | 96 | 67 | 76 | 0.96 | 0.58 |

**Table S3.** Summary of results for the US Method applied to each plant group using different administrative levels to define the location of a specimen. Italicized accuracies are significantly better than the default accuracy.

### *Specimen Count predictors*

The naïve Specimen Count method only used the number of specimens as the predictor. We counted the number of specimens as the number of georeferenced specimens present in the largest available dataset (the same as used for rCAT and ConR).

### *Number of species*

As each set of predictors had differing numbers of species with missing information, different numbers of species remained for each modelling approach (Table S2). Although this means that our comparison is not based on exactly the same data sets, this reflects the reality of using these approaches.

| Plant Group | Method | | | | |
| --- | --- | --- | --- | --- | --- |
|  | **Random forests** | **rCAT** | **ConR** | **Specimen Count** | **US Method** |
| Coffea | 105 | 105 | 105 | 105 | 105 |
| Legumes | 830 | 838 | 829 | 838 | 838 |
| MadPalms | 176 | 176 | 176 | 176 | 176 |
| Myrcia | 97 | 97 | 97 | 97 | 97 |
| OrchidsNG | 96 | 96 | 96 | 96 | 96 |
| Total | **1304** | **1311** | **1303** | **1311** | **1311** |

**Table S4.** The number of species with complete data available to use in each approach.

## **Execution of analysis**

We used the entire data set available to test rCAT, ConR, Specimen Count, and US Method.

Before training our random forests classifier, we removed highly correlated predictors and predictors with near-zero variance. We then centred all numerical predictors to their mean and scaled them by their standard deviation. We took 75% of our random forest data set as the training set and used repeated cross-validation (10-folds, 5 repeats) on this to tune the hyperparameters of the random forest classifier. We chose the best model based on the area under the receiver operator curve (ROC) and tested the performance of the best model on the remaining 25% of the data set. We measured predictor importance in the best random forest classifier as mean decrease in accuracy by predictor permutation on the out-of-bag samples. The *caret* package in R was used for all of this.

We calculated the threshold for Specimen Count by measuring the classification accuracy on the entire data set as the threshold was increased from 1 to 100 specimens, and then chose the threshold that maximised the accuracy. The best threshold was 12 georeferenced specimens (Fig S1).


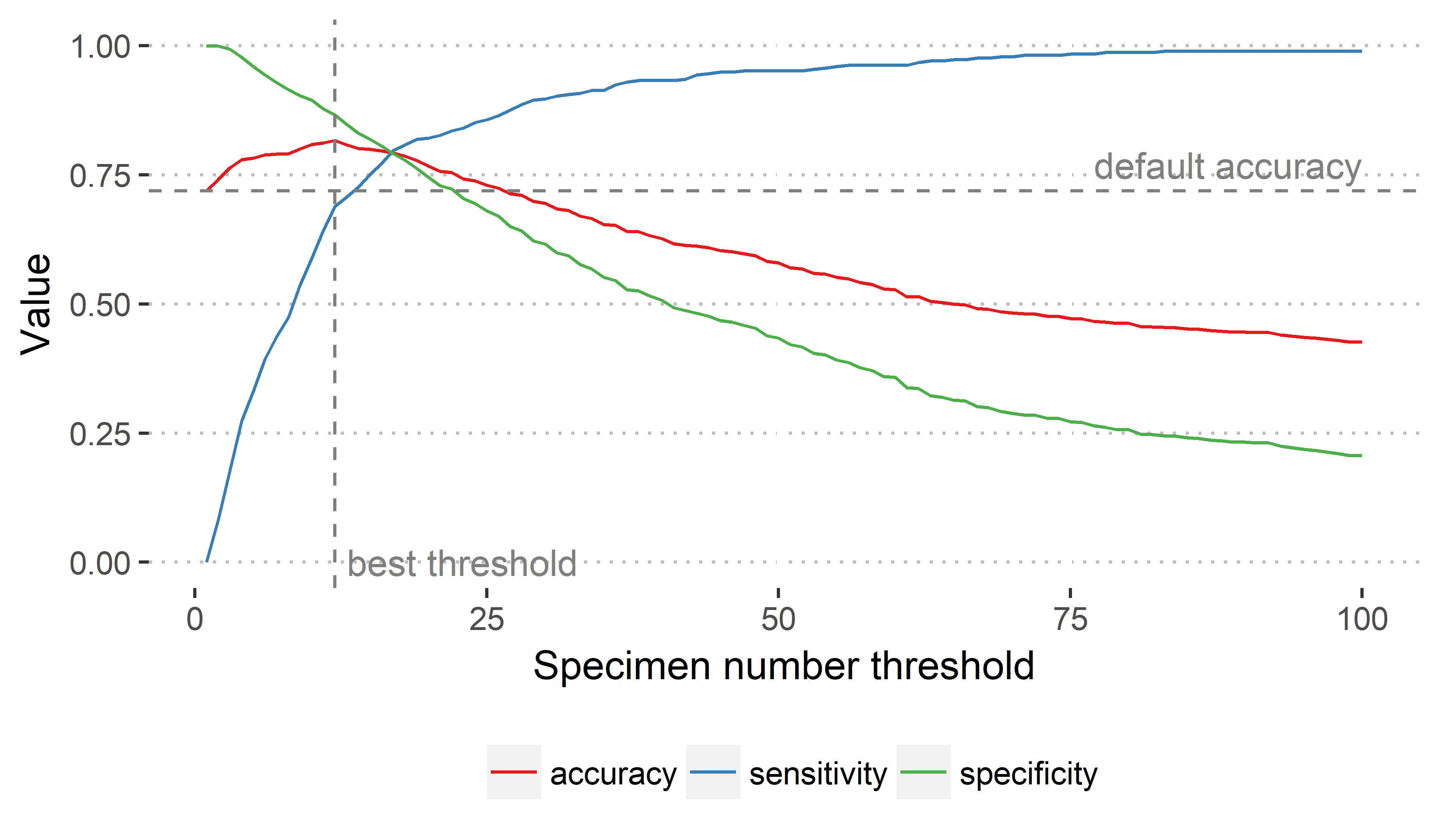


**Figure S1.** Accuracy, sensitivity, and specificity of classification by the Specimen Count approach using different thresholds. The best threshold, used in the approach comparison, is shown with a vertical dashed line.

## **Comparison of threat assessment**

We compared approaches based on their accuracy, sensitivity, and specificity. We also compared the accuracy of all approaches to the default accuracy, defined as the accuracy that could be achieved by classifying all species to the most common threat status in the data set.

We tested significance using Bayesian parameter estimation. For all parameter estimations, we chose flat conjugate priors to make computation easier. To estimate the accuracy of each approach, we chose a binomial likelihood for the number of correct classifications, which gave us the Beta distribution as the conjugate prior. The Beta distribution also had the benefit of being characterised by two parameters analogous to the number of correct and incorrect classifications, and outputting a number bounded between 0 and 1, as used for accuracy. We updated our flat prior with the number of correct and incorrect classifications and drew 10,000 samples from the resulting posterior.

To estimate the sensitivity and specificity of each approach, a multinomial likelihood was chosen to model the number of true positives, true negatives, false positives, and false negatives. This lead to our choosing flat Dirichlet prior for similar reasons as above. After updating the prior and drawing 10,000 samples from the posterior, we used the samples to calculate distributions for the sensitivity and specificity.

To test the significance of differences between approaches we first calculated the difference between the relevant posterior distribution samples, then assigned significance based on whether zero fell outside the 95% credible interval of the difference posterior distribution. We defined significant difference from the default accuracy as whether zero fell outside of the 95% credible interval of the accuracy posterior distribution for the approach in question.

We defined the credible interval of all distributions based on the highest posterior density interval.

|  | **Random Forests** | | **rCAT** | | **Specimen Count** | | **ConR** | | **US Method** | |
| --- | --- | --- | --- | --- | --- | --- | --- | --- | --- | --- |
|  | species | accuracy | species | accuracy | species | accuracy | species | accuracy | species | accuracy |
| **LC** | 205 | 0.961 | 832 | 0.956 | 832 | 0.883 | 825 | 0.828 | 832 | 0.778 |
| **NT** | 29 | 0.586 | 110 | 0.627 | 110 | 0.736 | 110 | 0.555 | 110 | 0.427 |
| **VU** | 32 | 0.656 | 121 | 0.669 | 121 | 0.479 | 120 | 0.683 | 121 | 0.727 |
| **EN** | 35 | 0.943 | 147 | 0.850 | 147 | 0.687 | 147 | 0.925 | 147 | 0.884 |
| **CR** | 25 | 0.960 | 101 | 0.980 | 101 | 0.941 | 101 | 1.000 | 101 | 0.990 |

**Table S5.** Accuracy of the different approaches by IUCN category, with the number of species in each test data set.


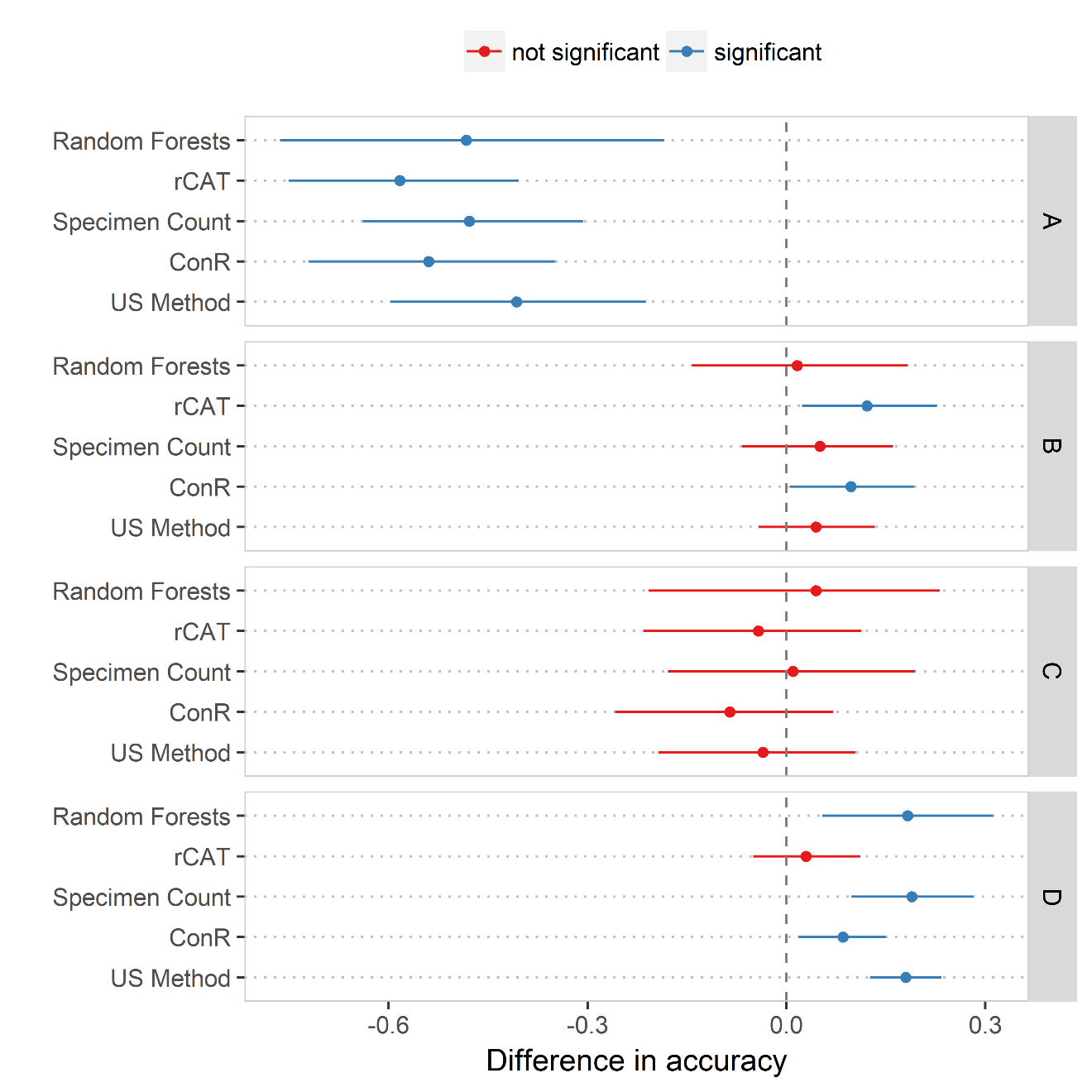


**Figure S2.**Comparison of the difference in classification accuracy between species assessed as threatened with and without an assessment by each IUCN Red List criteria for all approaches. Lines show the 95% credible intervals.


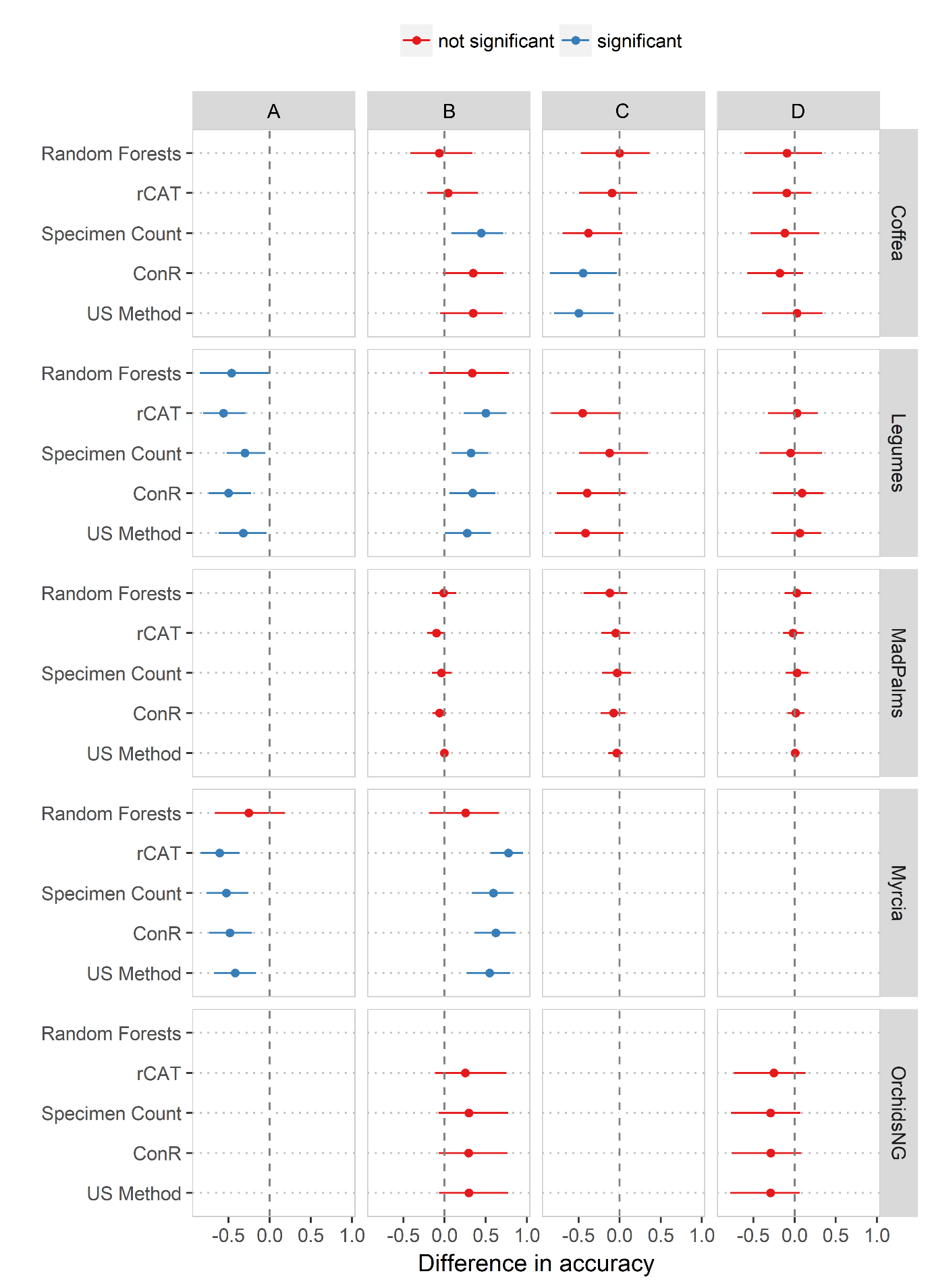


**Figure S3.**Comparison of the difference in classification accuracy between species assessed as threatened with and without an assessment by each IUCN Red List criteria for all approaches on each group of species. Lines show the 95% credible intervals.


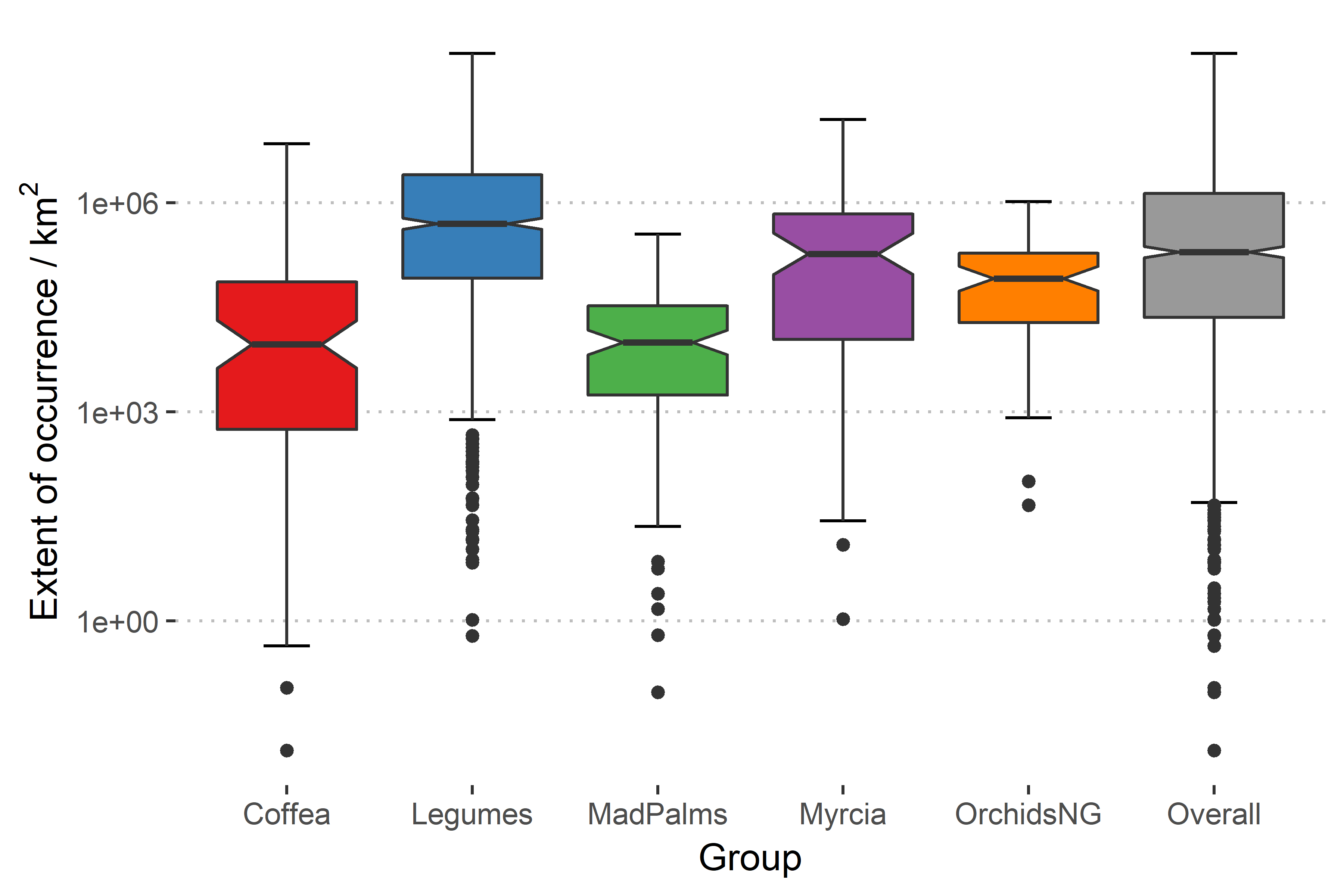


**Figure S4.**Boxplot showing mean Extent of occurrence for species in each group and in our dataset overall.

References

[1] Bland, L. M., Collen, B., Orme, C. D. L. & Bielby, J. 2015 Predicting the conservation status of data‐deficient species. *Conserv. Biol.* **29**, 250-259. (DOI:10.1111/cobi.12372).

[2] Cardillo, M., Mace, G. M., Jones, K. E., Bielby, J., Bininda-Emonds, O. R. P., Sechrest, W., Orme, C. D. L. & Purvis, A. 2005 Multiple causes of high extinction risk in large mammal species. *Science* **309**, 1239-1241. (DOI:10.1126/science.1116030).

[3] Hijmans, R. J., Cameron, S. E., Parra, J. L., Jones, P. G. & Jarvis, A. 2005 Very high resolution interpolated climate surfaces for global land areas. *Int. J. Climatol.* **25**, 1965-1978. (DOI:10.1002/joc.1276).

[4] Nordhaus, W. D. & Chen, X. 2016 Global Gridded Geographically Based Economic Data (G-Econ), Version 4. Palisades, NY: NASA Socioeconomic Data and Applications Center (SEDAC). See <http://doi.org/10.7927/H42V2D1C>.

[5] Center for International Earth Science Information Network - CIESIN - Columbia University. 2017 Gridded Population of the World, Version 4 (GPWv4): Population Count Adjusted to Match 2015 Revision of UN WPP Country Totals, Revision 10. Palisades, NY: NASA Socioeconomic Data and Applications Center (SEDAC). See <https://doi.org/10.7927/H4JQ0XZW>.

[6] Venter, O., Sanderson, E. W., Magrach, A., Allan, J. R., Beher, J., Jones, K. R., Possingham, H. P., Laurance, W. F., Wood, P., Fekete, B. M., et al. 2016 Global terrestrial Human Footprint maps for 1993 and 2009. *Sci. Data* **3**, 160067. (DOI:10.1038/sdata.2016.67).

[7] Dinerstein, E., Olson, D., Joshi, A., Vynne, C., Burgess, N. D., Wikramanayake, E., Hahn, N., Palminteri, S., Hedao, P., Noss, R., et al. 2017 An Ecoregion-Based Approach to Protecting Half the Terrestrial Realm. *BioScience* **67**, 534-545. (DOI:10.1093/biosci/bix014).

[8] Dauby, G., Stévart, T., Droissart, V., Cosiaux, A., Deblauwe, V., Simo‐Droissart, M., Sosef, M. S. M., Lowry, P. P., Schatz, G. E., Gereau, R. E., et al. 2017 ConR: An R package to assist large‐scale multispecies preliminary conservation assessments using distribution data. *Ecol. Evol.* **7**, 11292-11303. (DOI:10.1002/ece3.3704).

[9] Moat, J. 2017 rCAT: Conservation Assessment Tools. R package version 0.1.5. See <https://CRAN.R-project.org/package=rCAT>.

[10] Krupnick, G. A., Kress, W. J. & Wagner, W. L. 2009 Achieving Target 2 of the Global Strategy for Plant Conservation: Building a preliminary assessment of vascular plant species using data from herbarium specimens. *Biodivers. Conserv.* **18**, 1459-1474. (DOI:10.1007/s10531-008-9494-1).

[11] Global Administrative Areas v3.6 (2018). University of California, Berkely. See <http://www.gadm.org>.
